# Resin infiltrant for non-cavitated caries lesions: evaluation of color stability

**DOI:** 10.4317/jced.53110

**Published:** 2017-02-01

**Authors:** Matteo Ceci, Davide Rattalino, Matteo Viola, Riccardo Beltrami, Marco Chiesa, Marco Colombo, Claudio Poggio

**Affiliations:** 1 Department of Clinical-Surgical, Diagnostic and Pediatric Sciences - Section of Dentistry, University of Pavia, Pavia, Italy; 2 Department of Brain and Behavioural Sciences - Section of Statistic, University of Pavia, Pavia, Italy

## Abstract

**Background:**

The objective of this *in vitro* study was to evaluate the over time color stability of one resin infiltrant (Icon) upon exposure to staining solutions (coffee and wine) compared with one nano-hybrid sealant (Grandio Seal), one transparent fissure sealant with fluoride (Control Seal) and one nanofilled composite (Filtek Supreme XTE).

**Material and Methods:**

All materials were polymerized according to manufacturers’ instructions into silicon rings (height 1 mm; internal diameter 6 mm; external diameter 8 mm) to obtain specimens identical in size. The specimens were immersed in staining solutions at room temperature over a 28-day test period. The control samples have not been subjected to the staining process. A colorimetric evaluation according to the CIE L*a*b* system was performed by a blind trained operator at 7, 14, 21, 28 days of the staining process. Shapiro Wilk test and Kruskal Wallis ANOVA were applied to assess significant differences among different materials. Means were compared with Scheffe’s multiple-comparison test at the 0.05 level of significance.

**Results:**

In the case of all materials, immersion in solutions resulted in clinically perceivable color changes after 1 week (∆E < 3.3). Lowest CIE L* variation was registered for Control Seal and Grandio Seal both after 1 week and after 1 month, while Icon showed significantly higher variation (*P* < 0.05). Color coordinate CIE a* varied significantly more for Icon samples (*P* > 0.05). Color coordinate CIE b* varied similarly for all materials tested (*P* > 0.05).

**Conclusions:**

Immersion in coffee or red wine resulted in clinically perceivable color changes for all materials tested. Icon showed the highest color variations both after 1 week and 1 month. Icon can fix the initial esthetic problem associated with white spot lesions, but the resin may become more discolored than other materials over time.

** Key words:**CIE Lab, color stability, resin infiltrant.

## Introduction

Mineral loss, underneath an apparently intact surface layer, is the main characteristic of initial enamel caries lesions (1). Common side effect of orthodontic treatment with fixed brackets; these lesions are called white spots. Inadequate oral hygiene and salivary hypo function are other related risk factors (2). Cyclical imbalance between demineralization and remineralization, resulting from an acidic environment created by cariogenic bacteria, caused the subsurface enamel porosities of white spot lesions. Initial white spot lesions have an intact surface and are reversible (3). The first treatment for initial enamel caries is to promote lesion remineralization. Various treatments have been proposed: remineralization with topical agents such as fluorides (4) and amorphous calcium phosphates (CCP-ACP) (5), microabrasion (6), application of conventional resin bonding, sealants and minimal composite restoration (7).

Recently, resin infiltration represented an alternative approach to arrest initial carious lesions (8). Hinging on the modern philosophy of minimal invasive dentistry; this infiltration technique has become increasingly common in the past few years. The infiltration technique consist in using hydrophilic and low viscosity, light-cured resins that can penetrate white spot lesions subsurface micropores, inhibiting the diffusion pathway to cariogenic bacteria and their products and preventing further lesion progression (9). Several studies have demonstrated that resin infiltration lead to a significant increase of the micro hardness of initial enamel carious lesions (10,11). Recently some advantages of the resin infiltration technique have been highlighted by various Authors: mechanical stabilization of demineralized enamel, permanent obturation of superficial porous and deeply demineralized areas, preservation of sound hard substance, and arrest of lesion progress by increasing resistance to demineralization, minimized risk of secondary caries development and high patient acceptance (12). Compared to other treatment for white spot lesions, resin infiltration is less invasive than microabrasion and restorative treatments (13). The difference in refractive index (RI) between sound enamel and non-cavitated caries lesions cause the whitish appearance of white spot lesions. The resin infiltrant can eliminate the RI mismatch, filling the surface pores (8).

An infiltration resin showing good penetration characteristics (Icon, DMG, Hamburg, Germany) was recently available on the market. This resin was first developed in Germany, at the Charité University Hospital in Berlin, from *in vitro* studies on the penetration of resin into caries (14). This low-viscosity light-curing material has revealed good results in masking white spot lesions (15). The aim of infiltrating white spot lesions with Icon is to have highly esthetic results, not just immediately after treatment but for long-term effect. If the infiltrated carious lesions become stained over time, the esthetic outcome of the treatment would be compromised (16). Different studies have evaluated the color stability of artificial lesions infiltrated with Icon, after exposure to staining agents found in food and beverages, such as coffee, red wine or tea (17).

The objective of this *in vitro* study was to evaluate the over time color stability of one resin infiltrant (Icon) upon exposure to staining solutions (coffee and wine) compared with one nano-hybrid sealant (Grandio Seal), one transparent fissure sealant with fluoride (Control Seal) and one nanofilled composite (Filtek Supreme XTE). The null hypothesis of the study is that the color of Icon resin infiltrant would remain stable even when in contact with natural staining agents and that there would be no significant differences when compared with other restorative materials.

## Material and Methods

-Specimens’ preparation

In this study the over time color stability of one resin infiltrant (Icon) upon exposure to staining solutions (coffee and wine) was compared with one nano-hybrid sealant (Grandio Seal), one transparent fissure sealant with fluoride (Control Seal) and one nanofilled composite (Filtek Supreme XTE).  Table 1 shows details concerning the restorative materials used in this study.

**Table 1:**
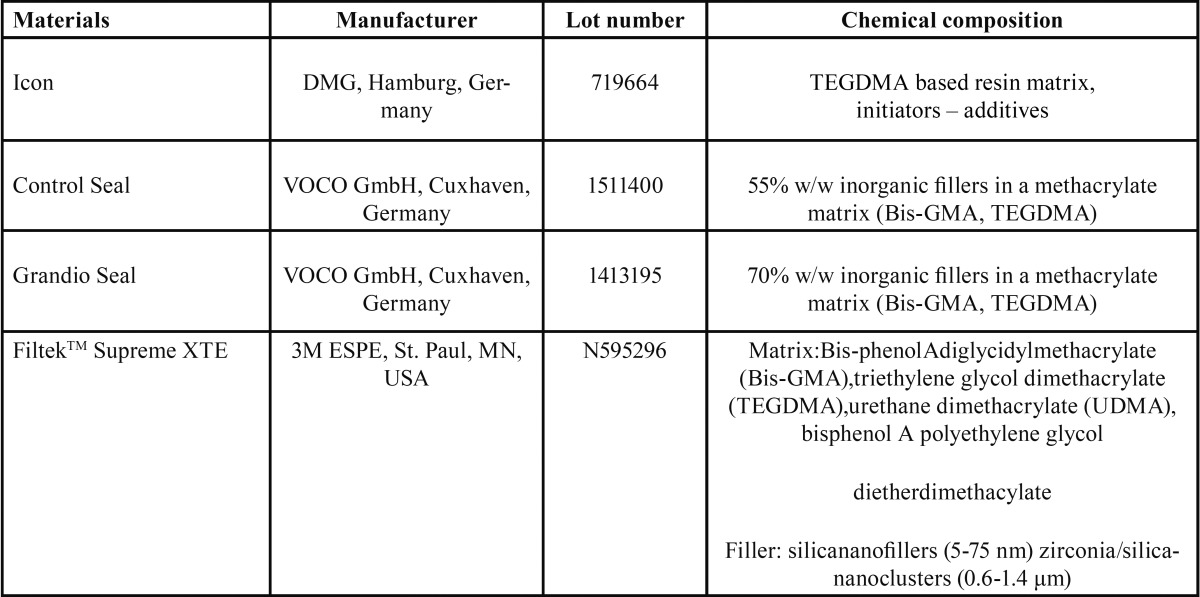
Characteristics of the materials tested in this study.

To obtain specimens identical in size, all the materials tested were polymerized according to manufacturers’ instructions into silicon rings (height 1 mm; internal diameter 6 mm; external diameter 8 mm). Cavities of these rings were slightly overfilled with material, covered by a Mylar strip (Henry Schein; Melville, NY), placed between 2 glass slides and polymerized for 40 seconds on each side using a polymerization unit (Celalux II, Voco, Cuxhaven, Germany). One light polymerization mode was used for each material - standard: 1000 mW/cm2 for 40 s. The intensity of the light was verified with a radiometer (SDS Kerr, Orange, CA). The light was placed perpendicular to the specimen surface at distance of 1.5 mm. With this procedure, 72 cylindrical specimens of each material were prepared. Specimens were stored in distilled water at 37°C during the whole experimentation period.

-Staining process 

The staining solutions used were: coffee (Nescafe Classic, Nestle, Vevey, Switzerland), red wine (Bonarda Tenuta Casa Re, Montecalvo Versiggia (PV), Italy) and physiological solution (negative control). The specimens were immersed in staining solutions at room temperature over a 28-day test period. The control samples have not been subjected to the staining process. Solutions were changed daily and put in vials with cover that prevent evaporation of staining solutions. Spectrophotometric analysis was made after 7, 14, 21 and 28 days the beginning of the experimentation. Before each measurement, the specimens were gently rinsed with distilled water and air-dried.

-Color testing

**Figure 1:**
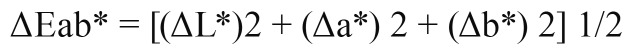
Formula.

A blind trained operator performed the colorimetric evaluation according to the CIE L*a*b* system at 5 experimental periods: immediately after light-polymerization and at 7, 14, 21, 28 days of the staining process. In order to simulate the absence of light in the mouth, the color of the specimens was measured against a black background with a spectrophotometer (SP820λ; Techkon Gmbh, Konig-Stein, Germany). All specimens were chromatically measured 4 times and the average values were calculated; then each color parameter for each specimens of the same shade was averaged. The CIE 1976 L* a* b* color system is used for the determination of color differences (19). The L* value refers to “lightness”; the higher is the L value, and it is the lightness (a value of 100 corresponds to perfect white and that of zero to black). CIE L* a* b* values are called the “chromaticity coordinates”; “a*” shows red color on positive values and green color on negative values; “b*” shows yellow color on positive values and blue color on negative values (18). The total color differences (ΔEab*) were calculated as follows (Fig. 1):

where L* is lightness, a* is green-red component (-a* = green; +a* = red) and b* is blue-yellow component (-b* = blue; +b* = yellow). A value of ΔEab* < 3.3 was considered clinically acceptable in the present study (19,22). Color measurements of the experimental groups were compared with those of the control group.

-Statistical analysis

Statistical analysis was performed using computer software (Stata 12.0, Stata Corp., Station College, TX). Descriptive statistics including the mean, standard deviation, median, minimum and maximum values were calculated for each color coordinate for all the groups. By applying the formula ∆Eab= (∆L2 + ∆a2 + ∆b2)1/2 it was possible to calculate ∆E and to compare the values before and after the staining immersion protocols. The distributions were assessed and found to be not normal (Shapiro-Wilk Test). Non-parametric Kruskal-Wallis one-way analysis of variance (ANOVA) by the factor of material was performed with the differences in color (∆E*ab) and three color coordinates (CIE L*, CIE a*, and CIE b*) between different immersion protocols in the specimen conditions such as before staining and after staining at the significance level of 0.05. Changes in color coordinates were calculated as “color coordinate of stained surfaces”. Means were compared with Scheffe’s multiple-comparison test at the 0.05 level of significance.

## Results

On the basis of one-way Kruskal-Wallis analysis of variance, staining processes with coffee and wine similarly influenced the color coordinates even after 1 week (*P* < 0.05), as reported in  Table 2.

**Table 2:**
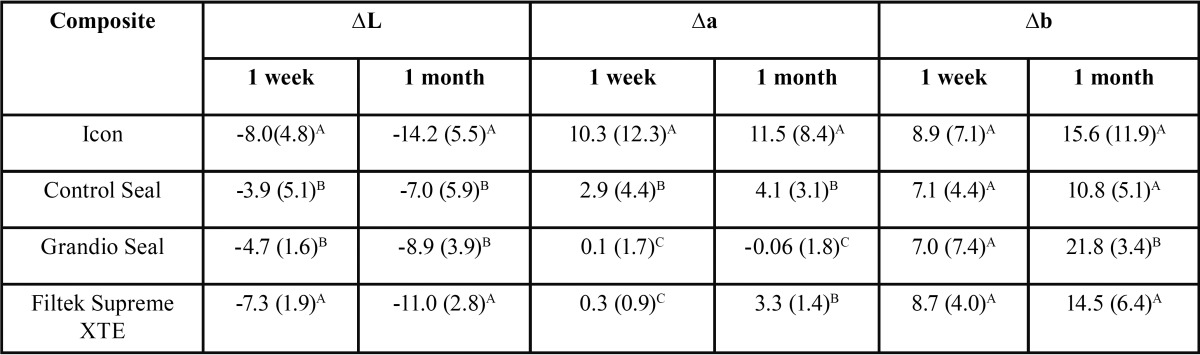
Mean difference in CIE L*, CIE a*, and CIE b* value (SD) for each composite after immersion protocol at 1 week and at 1 month in staining solutions. Different superscript letters indicate significant differences among materials.

**Figure 2:**
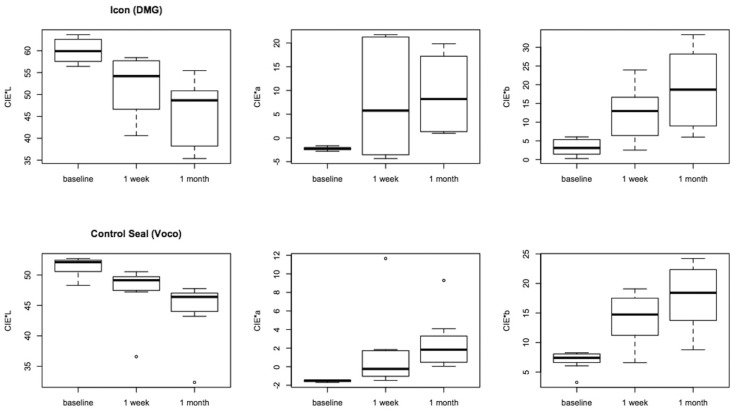
Box-plots for color coordinates CIE*L, CIE*a and CIE*b at different times for Icon and Control Seal. Bold horizontal line represents median.

**Figure 3:**
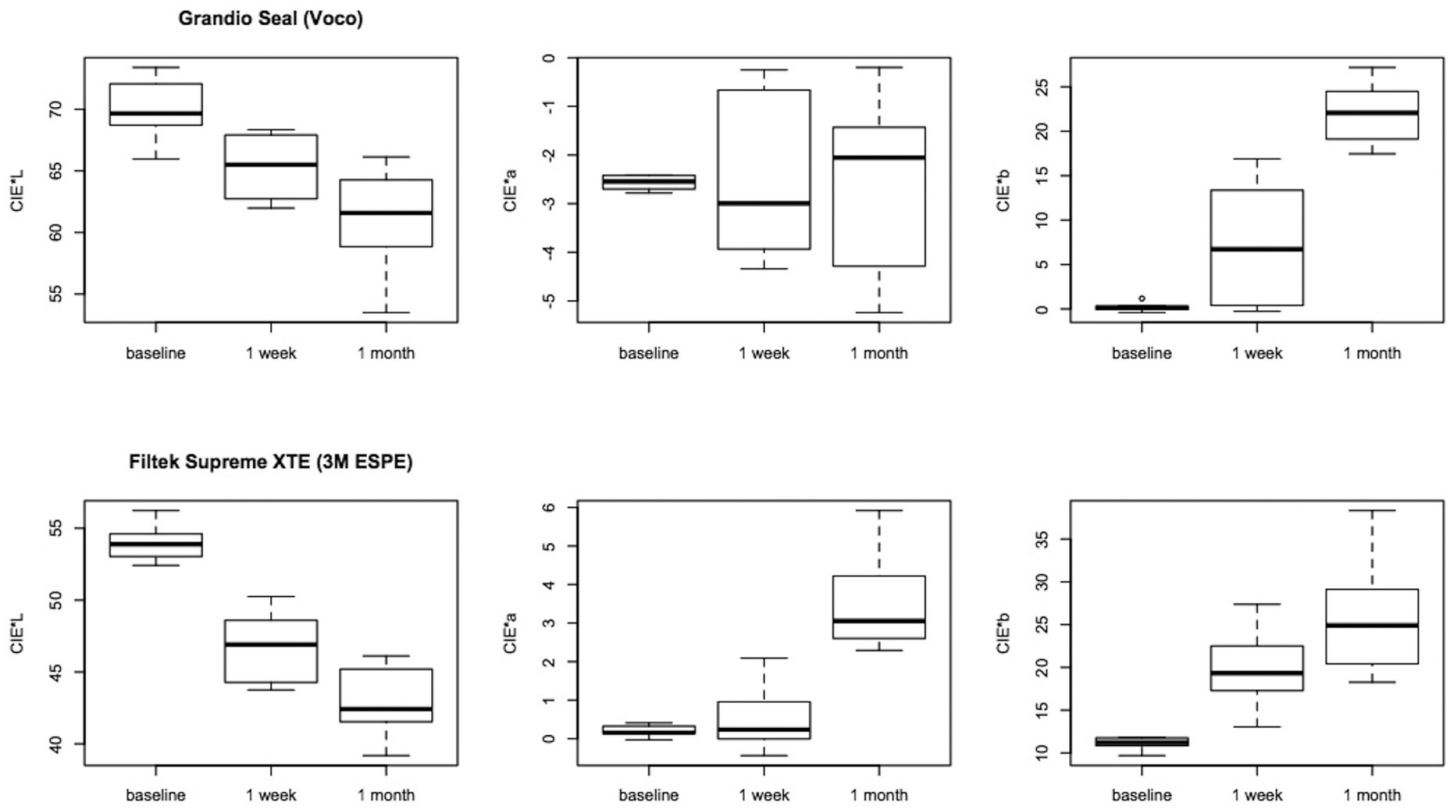
Box-plots for color coordinates CIE*L, CIE*a and CIE*b at different times for Grandio Seal and Filtek Supreme XTE. Bold horizontal line represents median.

Data regarding the two different staining solutions were merged in a single data set because we did not find significant differences between the two solutions over time. For each composite we could consider a larger number of specimens per group thus increasing the power of the analyses. Lowest CIE L* variation was registered for Control Seal and Grandio Seal both after 1 week and after 1 month, while Icon and Filtek Supreme XTE showed significantly higher variation (*P* < 0.05). Color coordinate CIE a* varied significantly more for Icon samples, while Control Seal showed lowest and comparable variation after 1 month (*P* > 0.05), as showed in figure 2. After 1 week color coordinate CIE b* varied similarly for all composite tested (*P* > 0.05) while after 1 month Grandio Seal showed the highest variation (*P* < 0.05), as showed in figure 3.

Based on repeated measures one-way ANOVA for the color difference between 1 week staining process and baseline and between 1 month staining process and baseline, brand of composite resin and staining (which was used as the ‘‘time’’ factor in repeated measures ANOVA) influenced the color difference (*P* < 0.05). Discoloration generally increased as the immersion period increased and, as reported in  Table 3, the value increased abruptly in the immersion period of 7 days to 1 month for Grandio Seal. Other materials showed steady and small increase by the immersion period. In the case of all materials, immersion in solutions resulted in clinically perceivable color changes after 1 week (∆E > 3.3).

**Table 3:**
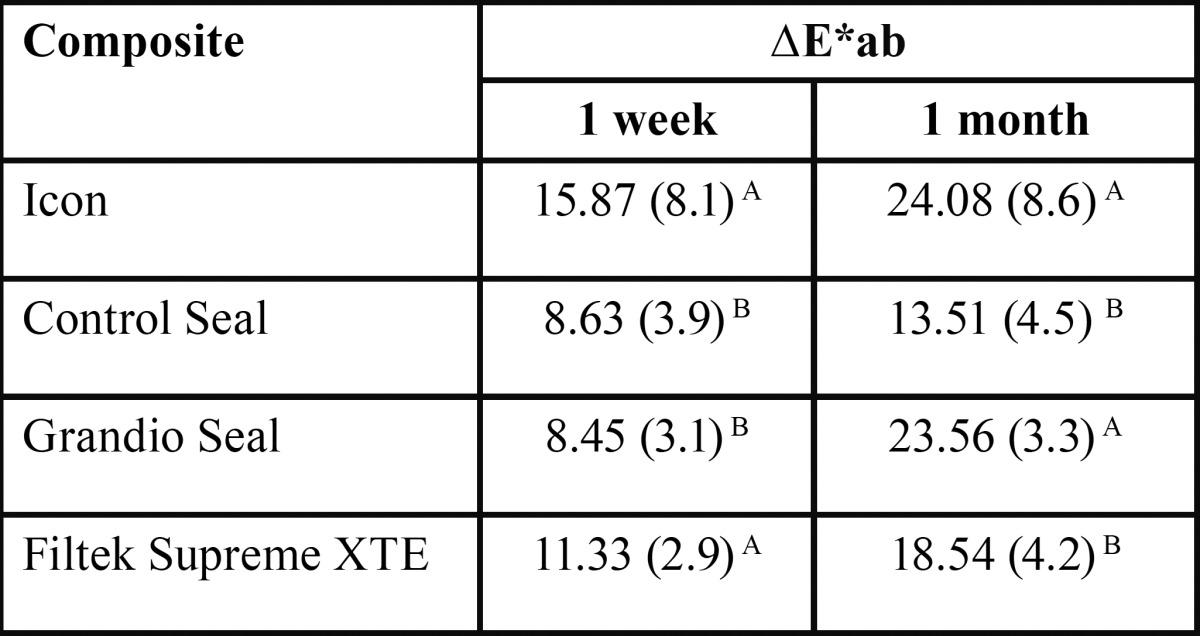
Mean color changes (SD) by staining after 1 week and 1 month. Different superscript letters in the same column indicate significant differences among materials.

## Discussion

The null hypothesis of this study, that the color of Icon would remain stable even when in contact with natural staining agents and that there would be no significant differences when compared with other restorative materials, was rejected. Icon resin infiltrant showed in fact significant color change (discoloration) over time.

Several studies proved the efficacy of the infiltration technique with Icon of non-cavitated caries lesions (8,9). However, there is also a need for this treatment to remain color-stable with time despite exposure to every day staining agents (19).

Over time color stability in the oral cavity is a fundamental property for esthetic restorative materials. Although improvements have been achieved during recent years, discoloration is still a problem (18). Visually and/or specific instruments can be used to assess color change of dental materials (20). The methodology used in the present study was in accordance with previous researches that used spectrophotometry and the CIE L*a*b* coordinate system, which is a widely used tool for dental purposes (21). Various studies reported the advantages of using the CIE L*a*b* coordinate system, such as its repeatability, sensitivity, and objectivity. This technique was chosen to evaluate the color variation (ΔE) because it is well suited for the determination of small color variations (21). ΔE values between 1 and 3 are perceptible to the naked eye and several Authors have stated that ΔE values greater than 3.3 are clinically unacceptable (18).

In this study, a long-term exposure with specimens stored for 28 days in different staining solutions was performed. According to Ertas *et al.* (22), this period should simulate around 2 years of clinical exposure to the staining agents (24 h *in vitro* corresponds to about 1 month *in vivo*), which is considered sufficient for long-term staining ability evaluation.

Common food substances could cause significant discoloration of composite resin materials (23). In this study, control group (physiological solution) maintain the same color, with no discoloration. Contrariwise, immersion in coffee or red wine resulted in clinically perceivable color changes after 1 week for all the materials tested (∆E > 3.3). Coffee has demonstrated in several studies a high capacity of staining composite resins and natural teeth (24). Discoloration by coffee was due to both adsorption and absorption of colorants. The absorption and penetration of colorants into the organic phase of materials were probably due to compatibility of the polymer phase with the yellow colorants of coffee (24). Also tannins, contained in red wine, possess a strong discoloration capacity. Red wine also contains alcohol; the sorption of alcohol molecules into the resin matrix could enhance the staining process, softening the composite resin surface (25). Despite various Authors reported that certain substances (e.g., coffee) may cause more severe staining than other (25); in this study the staining processes with coffee and wine similarly influenced the color coordinates.

In this study, although all the materials tested have demonstrated clinically perceivable color changes even after 1 week (∆E > 3.3); some differences have emerged between the various products tested. Icon reported significantly higher variation in CIE L* (lightness) and CIE a* (green-red component) values both after 1 week and 1 month of the staining process. Considering the total color differences (ΔEab*), Icon showed the highest values both after 1 week and 1 month. In general, the discoloration of all materials tested increased as the immersion period increased. However, while Icon, Filtek and Control Seal demonstrated steady and small increase by the immersion period; Grandio Seal reported an abrupt increase in ΔEab* values after 1 month. This sudden increase in discoloration values for Grandio Seal may be explained by a breakdown of the matrix components and/or by the over time degradation of the resin.

Resin monomers are the foremost common chemical components of composite restorative materials. Both acrylates and methacrylates monomers are vulnerable to water degradation (hydrolysis) of their ester groups (26). The spacer of the monomer keeps functional and polymerizable groups separated; because of its hydrophilicityit may cause water uptake, which leads to higher hydrolysis susceptibility of the monomers as well as discoloration of the cured resin (26). According to Sideridou *et al.* (27), TEGDMA has the highest water sorption capability, followed by BisGMA and by UDMA. Although in different proportion, all the materials tested in this study present in their chemical compositions the TEGDMA resin monomer. This may explain the clinical unacceptable color change (∆E > 3.3) of all the products tested. However, TEGDMA is the main component of Icon. TEGDMA demonstrated in fact the best ability to infiltrate deep into the non-cavitated caries lesion. Paris *et al.* demonstrated a higher penetration coefficient into the lesion for a resin consisting mainly of TEGDMA (10). Nevertheless, TEGDMA has the highest water sorption rate, which causes resin discoloration. Therefore, in this study Icon (composed mainly of TEGDMA) became more discolored after storage in staining solutions as compared with other materials. This is also increased due to the fact, that Icon does not include any filler. Icon is pure resin and any pure resin will be stained quickly.

Our results are in accordance with recent studies, which demonstrated that Icon presents significant alteration of color after staining processes (17,28). Similarly to our study, Borges *et al.* reported significantly higher discoloration for Icon infiltration resin compared to all other tested groups (17). However, there are also clinical data available, investigating the long time color stability of infiltrated vestibular tooth surfaces. Two of these recent studies reported that, in the oral environment, Icon’s masking effects are stable and that there is no significant discoloration. These Authors also mentioned that polishing will remove any staining (29,30).

## Conclusions

Results obtained from the present study could be of clinical relevance. Clinicians are provided with information on the staining potential of esthetic restorative materials when exposed to natural staining agents. All the materials tested showed significant color alterations after exposure to staining dyes. It is speculated that while Icon can fix the initial esthetic problem associated with white spot lesions, the resin may become more discolored than other materials over time, especially when the patient habitually consumes teeth-staining food and beverages. Nevertheless, this has been investigated on pure Icon discs. Icon will not be available in the oral environment in pure form. In the clinical setting Icon will only be a part of a stained tooth area, which will also include tooth structure. Also, polishing of the infiltrated tooth area is mandatory in the clinical situation to remove the surface layer, which stains strong over time. In conclusion, more *in vivo* studies are needed to assess the real staining potential of Icon resin infiltration technique.
